# Combining Healthcare-Based and Participatory Approaches to Surveillance: Trends in Diarrheal and Respiratory Conditions Collected by a Mobile Phone System by Community Health Workers in Rural Nepal

**DOI:** 10.1371/journal.pone.0152738

**Published:** 2016-04-25

**Authors:** David J. Meyers, Al Ozonoff, Ashma Baruwal, Sami Pande, Alex Harsha, Ranju Sharma, Dan Schwarz, Ryan K. Schwarz, Deepak Bista, Scott Halliday, Duncan S. R. Maru

**Affiliations:** 1 Possible, Bayalpata Hospital, Sanfebagar-10, Achham, Nepal; 2 Harvard T. H. Chan School of Public Health, Department of Health Policy and Management, Boston, Massachusetts, United States of America; 3 Boston Children’s Hospital, Center for Patient Safety and Quality Research, Boston, Massachusetts, United States of America; 4 Harvard Medical School, Boston, Massachusetts, United States of America; 5 United Nations Population Fund, Kathmandu, Nepal; 6 Medic Mobile, San Francisco, California, Unuted States of America; 7 Brigham and Women’s Hospital, Department of Medicine, Division of Global Health Equity, Boston, Massachusetts, United States of America; 8 Boston Children’s Hospital, Department of Medicine, Division of General Pediatrics, Boston, Massachusetts, United States of America; 9 University of Washington, Henry M. Jackson School of International Studies, Seattle, Washington, United States of America; 10 Harvard Medical School, Department of Medicine, Boston, Massachusetts, United States of America; University College London, UNITED KINGDOM

## Abstract

**Background:**

Surveillance systems are increasingly relying upon community-based or crowd-sourced data to complement traditional facilities-based data sources. Data collected by community health workers during the routine course of care could combine the early warning power of community-based data collection with the predictability and diagnostic regularity of facility data. These data could inform public health responses to epidemics and spatially-clustered endemic diseases. Here, we analyze data collected on a daily basis by community health workers during the routine course of clinical care in rural Nepal. We evaluate if such community-based surveillance systems can capture temporal trends in diarrheal diseases and acute respiratory infections.

**Methods:**

During the course of their clinical activities from January to December 2013, community health workers recorded healthcare encounters using mobile phones. In parallel, we accessed condition-specific admissions from 2011–2013 in the hospital from which the community health program was based. We compared diarrhea and acute respiratory infection rates from both the hospital and the community, and assigned three categories of local disease activity (low, medium, and high) to each week in each village cluster with categories determined by tertiles. We compared condition-specific mean hospital rates across categories using ANOVA to assess concordance between hospital and community-collected data.

**Results:**

There were 2,710 cases of diarrhea and 373 cases of acute respiratory infection reported by community health workers during the one-year study period. At the hospital, the average weekly incidence of diarrhea and acute respiratory infections over the three-year period was 1.8 and 3.9 cases respectively per 1,000 people in each village cluster. In the community, the average weekly rate of diarrhea and acute respiratory infections was 2.7 and 0.5 cases respectively per 1,000 people. Both diarrhea and acute respiratory infections exhibited significant differences between the three categories of disease rate burden (diarrhea p = 0.009, acute respiratory infection p = 0.001) when comparing community health worker-collected rates to hospital rates.

**Conclusion:**

Community-level data on diarrhea and acute respiratory infections modestly correlated with hospital data for the same condition in each village each week. Our experience suggests that community health worker-collected data on mobile phones may be a feasible adjunct to other community- and healthcare-related data sources for surveillance of such conditions. Such systems are vitally needed in resource-limited settings like rural Nepal.

## Background

Disease surveillance is a vital component of a functioning healthcare system [1]. Ambulatory and emergency department data collected during the routine course of clinical care have been used effectively to detect disease clusters, such as influenza syndromes [2–4]. Surveillance is of particular concern in resource-limited settings where constrained financial and human resources affect the ability of healthcare systems to respond to changes in disease patterns and supply needs [5–7]. Prospective analytic methods have demonstrated utility as surveillance tools, but are hindered in resource-limited settings by the lack of reliable, real-time data [8–10].

With the rise of information and communication technologies, a number of interventions have targeted community- or crowdsourced-data to supplement traditional healthcare facility data for surveillance [11,12]. Social media postings [13–15] and internet search engine queries [16] have been used with varying degrees of success to track disease trends. Recent efforts such as InfluenzaNet [17] and Health Map [18] have attempted to crowdsource health information from participants around the world in order to better evaluate disease trends. Recent studies have found modest success in correlating trends between information collected from these new sources and disease occurrence indicating that such approaches may be viable and of public health and clinical significance [19–21]. While some recent studies have attempted to look at participatory surveillance using new electronic media in middle income countries, self-reported data can have limitations and few studies have taken places in settings of rural poverty [22] [23,24].

The concurrent expansion of mobile phones and community health workers (CHWs) offers new opportunities for surveillance in resource-limited areas. While some studies have sought to evaluate the efficacy of CHW surveillance systems through describing their activities and their use in tracking outcomes such as child nutrition [25–28], relatively few studies have investigated the feasibility of CHW-based surveillance systems using mobile phone applications [29]. Nepal, owing to a resource-constrained public sector healthcare system, geographic isolation, and high levels of poverty, currently has weak surveillance systems. This places particular importance on the development of affordable solutions for rural data collection [30,31]. Coupling CHW-collected data on mobile phones with healthcare facility data could combine the strengths of community-based participatory approaches with the accuracy of healthcare facilities data.

Here, we assess how closely the clinical data collected on a daily basis by CHWs on mobile phones during routine care in rural Nepal reflects diarrhea and acute respiratory infection (ARIs) patterns in a district hospital’s catchment area population. To evaluate this method of data collection, we compare trends between the mobile-collected data and hospital-based rates. We choose these syndromes both because they are widespread and because they are of particular relevance to rural healthcare systems. Diarrheal disease is the seventh leading cause of death worldwide and is responsible for killing over 1.5 million people annually [32]. ARIs, the fourth leading cause of death worldwide, account for over 3.1 million deaths annually [32].

## Materials and Methods

### Study Site

We conducted the study in collaboration with *Possible*, a non-profit healthcare company working in a public-private partnership with the Nepal Ministry of Health to deliver healthcare within the public sector in Achham District. Their hub of operations is the government-owned Bayalpata Hospital, in Achham.

Achham District is in Nepal’s Far-Western Development Region and is home to approximately 257,000 citizens [33]. The district is one of the most impoverished regions in South Asia, and the second-poorest district in Nepal [34]. Nepal’s national neonatal, infant, and under-five mortality rates are 33, 46, and 54 deaths per 1,000 live births, respectively. The Far-Western Development Region is home to the highest under-five mortality rate in Nepal, at 82 (one in 12) deaths per 1,000 live births [35].

Administratively, as with other districts in Nepal, Achham is divided into Village Development Committees (hereafter, village cluster) of approximately 1,000–5,000 people. Each village cluster is further divided into nine villages, which are the smallest administrative divisions in Nepal. The Government of Nepal operates a national Female Community Health Volunteer (FCHV; hereafter, community health workers or CHWs) network, with each village being allocated 1–2 volunteers. The volunteers work on improving maternal and child health outcomes, providing referrals to hospitals, and providing basic clinical services in the community.

*Possible* expanded upon this model in 14 village clusters (8 village clusters during the course of study) in the area surrounding Bayalpata Hospital. They accomplished this through providing additional training to the CHWs, a stipend for their work, and supervision by a Community Health Worker Leader (CHWL) in each village cluster. In their program, they use the term “community health worker” to emphasize that they are considered professional workers rather than volunteers within this structure.

### Ethics Review Board

The study received IRB approval from the Brigham and Women’s Hospital IRB (Boston, Massachusetts, USA; #2013P000709) and from the Nepal Health Research Council (#79/2012). Written informed consent was received from the patients to collect the community healthcare encounter data and written informed consent was received from the CHWs to use that data in this research study.

### Community Health Data Collection

CHWs regularly visited families within their assigned village. If they identified a community member exhibiting any symptoms, or if community members sought them out with any condition, they recorded on paper the condition, the date the visit occurred, how long since they last saw that patient, the age and gender of the patient, and what treatments or referrals they provided to the patient to address their condition. The CHWs were trained in the identification of common diseases and injuries by clinicians at Bayalpata Hospital, and through a national training curriculum. Twice weekly, these CHWs submitted these data to their CHWL. *Possible*’s CHWL then used a mobile phone-based data collection application to enter data. The CHWLs were trained by *Possible* to ensure consistency between reporting methods. The goal of the application was to aid the CHWLs in the accurate collection of the data, and to improve the timelier reporting and response to data trends.

The interface was designed in partnership with the non-profit technology company Medic Mobile [36], to facilitate triage and to coordinate follow-up care. For data collection by CHWs, Medic Mobile’s Muvuku software was designed to use Nepali script (Devanagri). This software ran on a parallel SIM card system (turboSIM), which allowed the data entry application to run on regular mobile phones (Samsung 1080t) by inserting the parallel SIM card between the phone and its original SIM card. The original SIM cards provided to the CHWLs were for a cellular service provider that had unreliable service for the region, yet when this was discovered a different SIM card was installed in each phone that did not have connectivity issues. The software used the native menu of the phone and contained validation parameters that limited digits or characters for each field to improve data quality. The application was capable of storing completed forms for later submission if the phone was out of reach of a mobile network. In the case of unreliable electricity, all of the phones had an accompanying solar charger that could replenish the phones’ batteries. Data were uploaded to Medic Mobile’s custom designed server, Kujua. The software program enabled CHWLs to track patients by recording their home location, the condition for which they were being followed, and the next action steps in their care. The system kept patient data secure in compliance with HIPPA regulations in the United States.

CHWLs were trained in the use of the software by a team from Medic Mobile. All CHWs received additional training in data collection and the provision of community healthcare once every three months. Bayalpata Hospital informatics staff members were also trained in the use of the Kujua database software.

### Clinical Data Collection

Bayalpata Hospital received patients both referred by CHWs and who presented without referral. Clinicians recorded all clinical data on paper forms and then trained data entry specialists entered these data into a Microsoft Access database.

We abstracted three years of data from 2011–2013 from Bayalpata Hospital’s Outpatient and Emergency Departments in order to look at trends over time. As annual hospital rates of diarrhea and ARI were similar, we only included data from 2013 in this analysis. Emergency Department data from January 2011 were not available as electronic data entry for that department did not begin until February 2011. We limited these data to records with Nepal Health Management Information System (HMIS) codes representing conditions that a CHW may label as either diarrhea or ARI (Table 1). 99.4% of all encounters had HMIS codes (99.3% of all Outpatient Department encounters and 99.7% of all Emergency Department encounters). These data were matched to the village cluster of patient residence, and we calculated weekly rates per 1,000 village cluster residents over the three-year study period. We then calculated the same outcomes from the 2013 hospital data alone. Population data for each village were taken from the 2011 Nepal National Census [33].

**Table 1:** HMIS Codes for Diarrhea and ARI in Bayalpata Hospital Data System.

Condition	HMIS code
Diarrhea	
Acute gastro enteritis	201
Amoebic dysentery/Amebiasis	202
Bacillary dysentery/Shigellosis	203
Presumed non-infectious diarrhea	204
Cholera	205
Intestinal worms	206
ARI	
Lower respiratory tract infection	450
Upper respiratory tract infection	451
Pneumonia	452
Severe pneumonia	453
Bronchitis	454
Influenza	456
Bronchiolitis	461
Croup	562

### Software

Medic Mobile’s Muvuku software was used for data collection by the CHWLs. These data were aggregated in Medic Mobile’s Kujua database tool and exported to Microsoft Excel 2013. Data management and statistical analysis were conducted in SAS version 9.2 (Cary, NC) and STATA 13.0 (College Station, TX)

### Statistical Analysis

We used several methods to compare the trends between the community- and hospital-collected data streams. We used a square root transformation of each rate for distributional reasons, and plotted transformed weekly rates of each condition during 2013.

We categorized the transformed community health rates by tertiles: low, medium, and high. We conducted an ANOVA comparison of the square root transformed hospital rates across the categories of CHW-reported disease activity. We similarly categorized hospital rates, cross-tabulated these categories with the community health data categories, and calculated the proportion of week-village combinations that had concordant categories.

## Results

### Basic Descriptive Data

The community health program collected a total of 17,435 encounters during 2013. Of these, there were 2,710 reported cases of diarrhea during the study period and 373 cases of ARI collected by CHWs (Table 2). We display the mean weekly rates of diarrhea and ARI reported by Bayalpata Hospital and the CHWs in Table 2. ARI primarily affected children while diarrhea was reported more frequently among adults.

**Table 2:** Basic Description of the Study Sample Stratified by Type of Condition.

	Diarrhea	ARI
**Count of Community Reported Cases**	2710	373
**% Female**	65.2%	41.3%
**Mean Age**	23.6 (SD 19.8)	2.3 (SD 4.0)
**Mean Community Rate**^a^	2.7 (95% CI: 2.3–3.0)	0.46 (95% CI: 0.4–0.6)
**Mean Hospital Rate**^a^	1.5 (95% CI: 1.4–1.7)	3.29 (95% CI: 3.0–3.6)

^a^Rates calculated weekly per village cluster per 1,000 people

### Clinical vs. Community Rates of Disease

**Fig 1:**
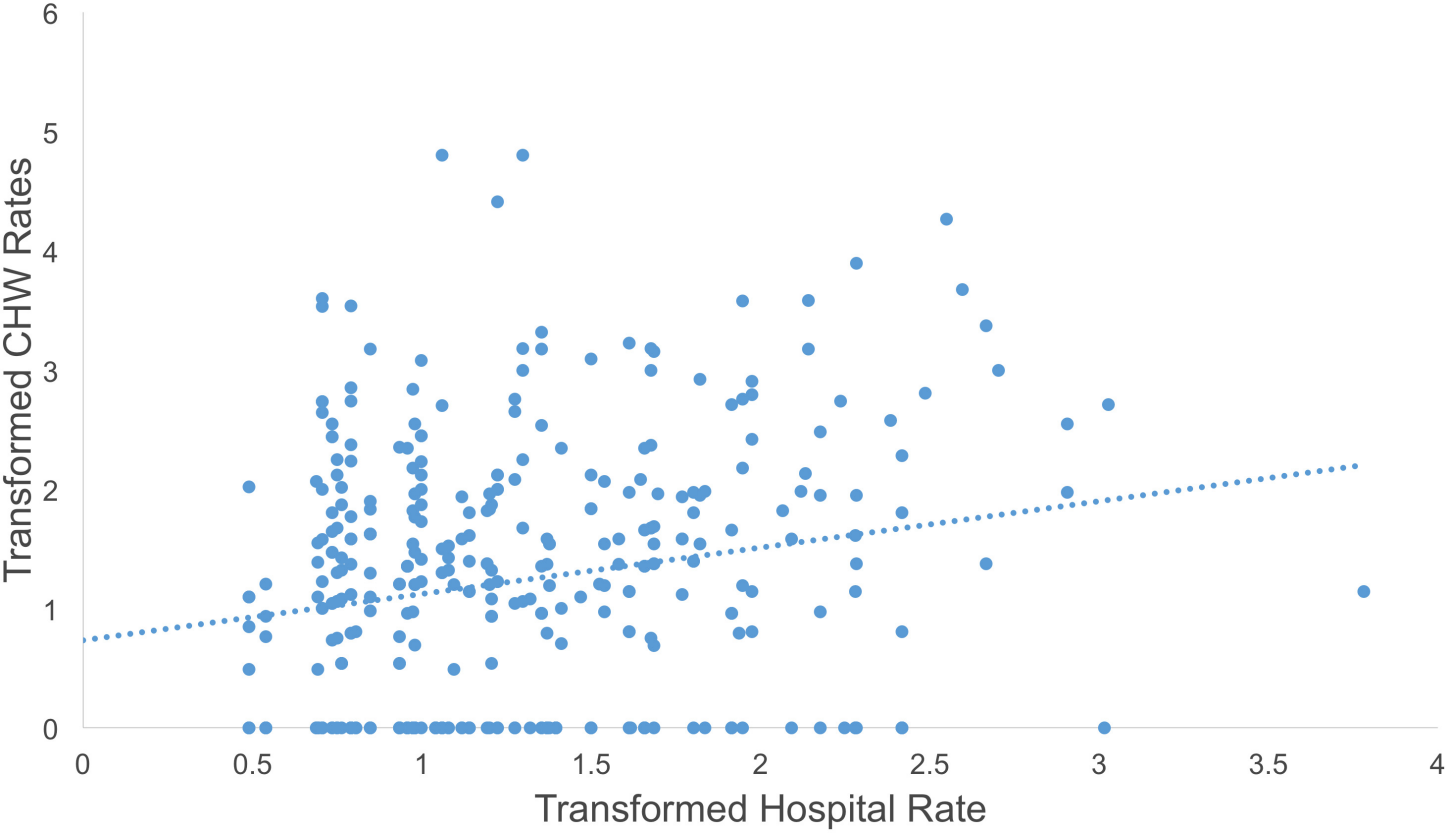
Scatter plot of hospital diarrhea rate per 1,000 people against community health diarrhea rate per 1,000 people.

**Fig 2:**
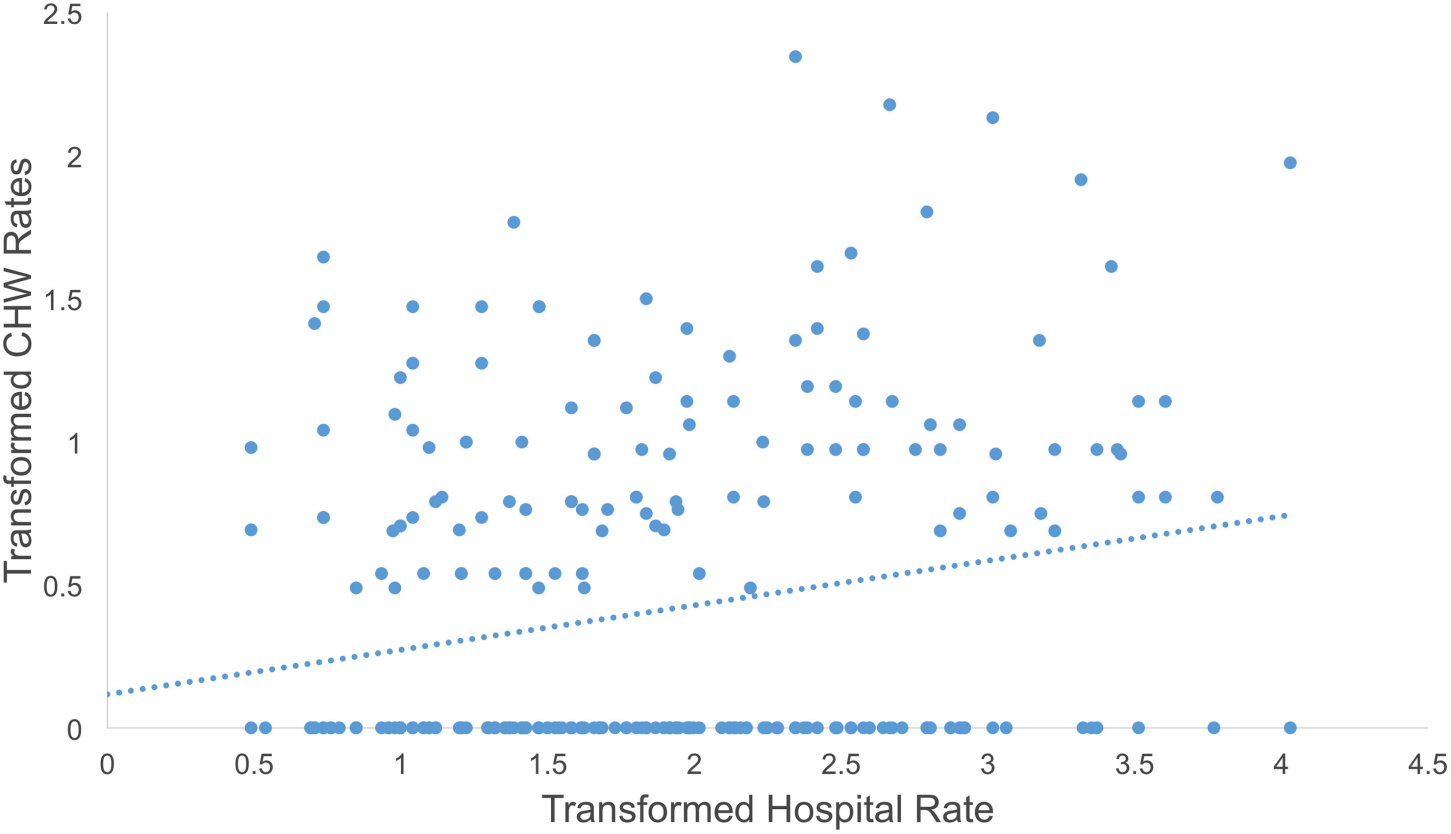
Scatter plot of hospital ARI rate per 1,000 people against community health ARI rate per 1,000 people.

Condition rates for both the hospital and community health streams were skewed to the right. We square root transformed them in order to achieve the assumption of normality. When comparing the transformed weekly rates of diarrhea and ARI in the community and the hospital using scatter plots, there appeared to be a direct relationship between both rates in each plot despite a large degree of clustering around zero during weeks when there were no reported cases of diarrhea or ARI from either data stream (Figs 1 and 2).

**Fig 3:**
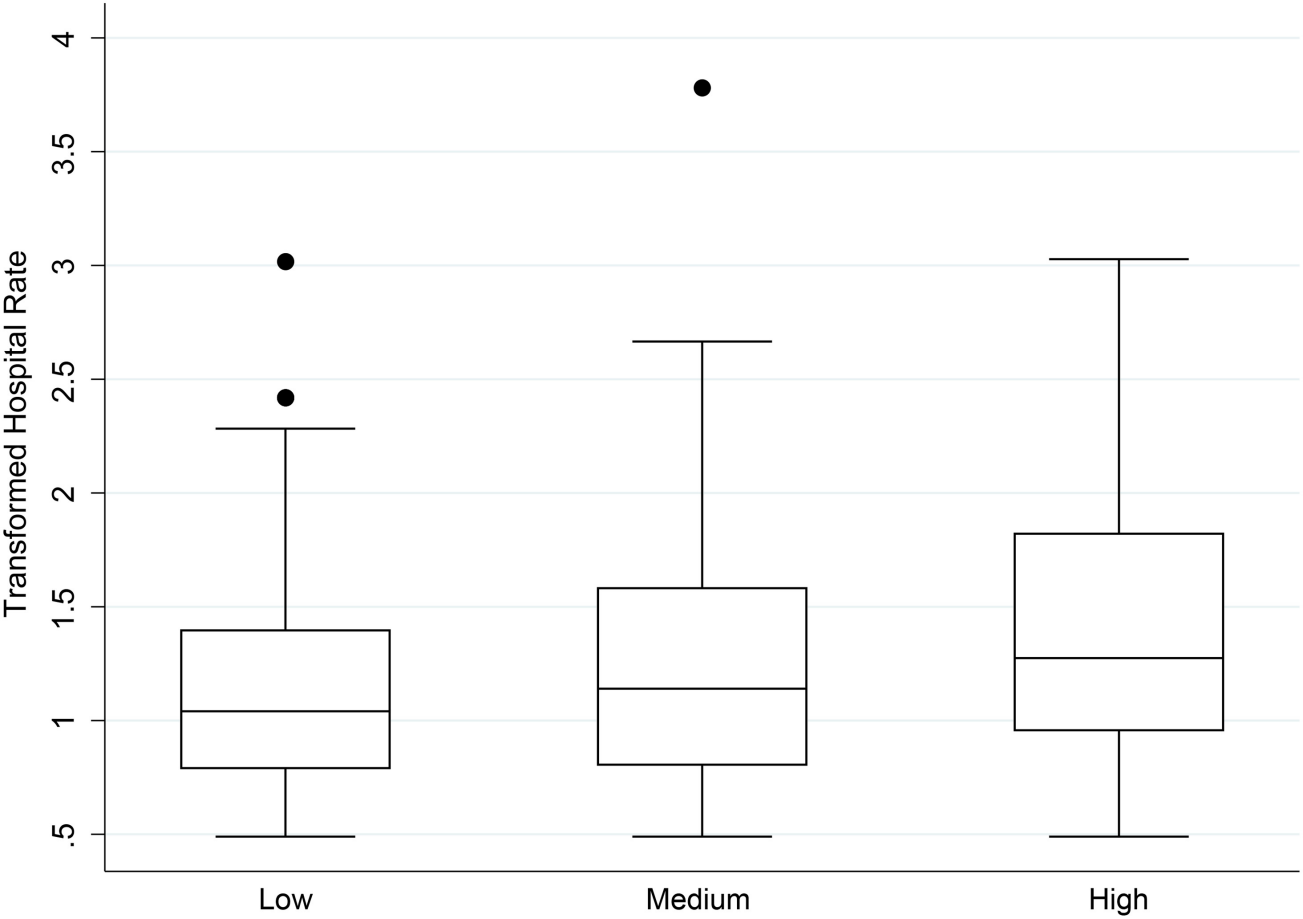
Box plots of hospital diarrhea rates per week per village cluster per 1,000 people by community health rate categories^a^. Dots represent outliers in the data.

**Fig 4:**
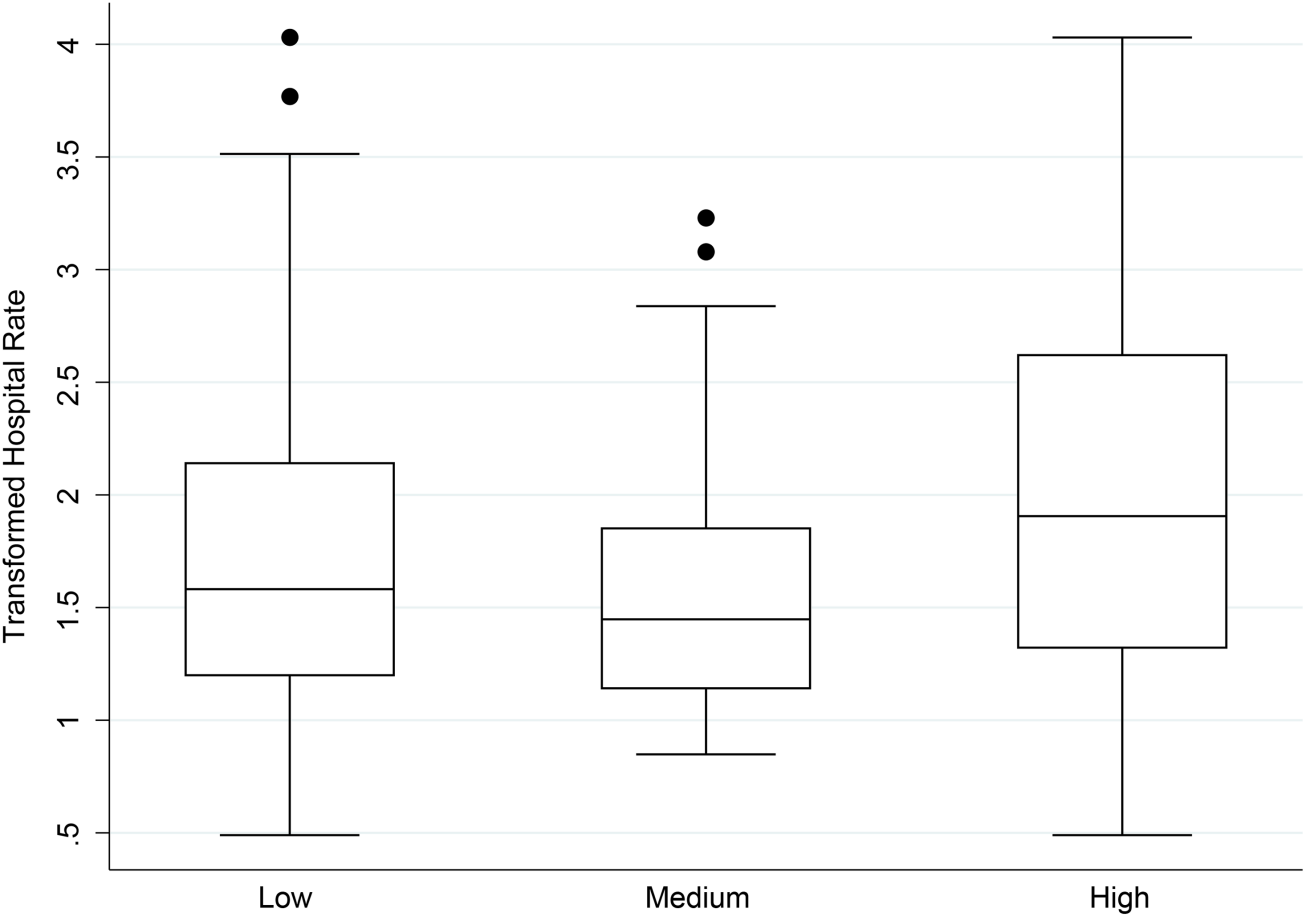
Box plots of hospital ARI rates per week per village cluster per 1,000 people by community health rate categories^a^. Dots represent outliers in the data.

When using ANOVA, both diarrhea and ARIs exhibited significant differences between the three different classification levels of cases (diarrhea p = 0.009, ARI p = 0.001) (Tables 3 and 4, Figs 3 and 4). For diarrhea, post-hoc comparisons revealed significant differences between low versus high (p = 0.001) and medium vs. high (p = 0.04). For ARI, the only significant difference was between low versus high (p = 0.01). All p-values for pairwise comparisons were Bonferroni-adjusted for multiple comparisons.

**Table 3:** Diarrhea Hospital Rates Versus CHW Reported Rates.

		Community Health Level					
	Level	Low	Medium	High	Total		
Hospital Level	Low	54	45	30	129	Diagonal:	148
	Medium	42	43	41	126	Concordance:	0.40
	High	28	36	51	115		
	Total	124	124	122	370		

**Table 4:** ARI Hospital Rates Versus CHW Reported Rates.

		Community Health Level					
	Level	Low	Medium	High	Total		
Hospital Level	Low	61	7	24	92	Diagonal:	113
	Medium	72	9	32	113	Concordance:	0.33
	High	51	43	43	137		
	Total	184	59	99	342		

Tables 3 and 4 show the community health rate categories against the hospital rate categories for the three-year hospital averages of diarrhea and ARI respectively. The concordance statistic for diarrhea was 40.0% (95%CI 35.0–45.2%) and the concordance statistic for ARI was 33.3% (95%CI 28.1–38.3%).

## Discussion

We find that data collected using mobile phones from rural CHWs correlates modestly with data collected at a district-level hospital for conditions such as diarrhea and ARI. This contributes to a growing body of literature that mobile phone-based surveillance systems may have the potential to be efficacious in tracking community disease trends in low-income [29,37] and geographically-isolated locations [38,39]. The similarity in trends could be a result of the CHWs successfully referring patients to the hospital to receive care. It may also reflect the ability of CHW-collected data to be responsive to disease dynamics in the community in near real-time. In most weeks for both diarrhea and ARI, there were higher rates in the community health data. It is notable that community-reported diarrhea rates were higher overall than those captured by hospital visit data. This may suggest that CHWs see lower acuity or quickly resolving cases, that community members face barriers to accessing care at the hospital, or most probably, both.

The greater number of women with reported ARIs and diarrhea may be due to increased access to the CHWs as many of the men in these communities work outside of the district for much of the year [40]. This trend would thus be expected given the patterns of migrant labor in Achham [35] and may suggest that CHW data accurately reflects population dynamics. It is also possible the CHWs make less frequent contact with males due to the timing of the visits during the workday, or due to social barriers related to gender.

The relatively low concordance statistics between hospital and community data could be driven by substantial overlap among all three categories, as illustrated in the boxplots (Figs 3 and 4). If CHWs are able to help provide an early warning system, the district-level hospital could be better prepared to deal with potential outbreaks.

There are several important limitations of this study. The first is that hospital visit data does not represent a gold standard for population health surveillance, particularly in populations with limited access to healthcare. In this context, a more comparable dataset may have been the case rates at local healthcare facilities that routinely care for mild acute illnesses such as those chosen for the current study. These data are not easily accessible, however, given the paper-based records and the degree of data aggregation (i.e. monthly or quarterly caseloads) in the government reporting system. Additionally, the CHWs only report the frequency of cases and not their severity, while the hospital might only see the most severe cases. This might create a discrepancy between the number of cases reported at community and hospital levels.

Given the absence of a gold standard comparator, it is important to consider the potential for systematic biases at various points during data collection by CHWs as well in hospital-based data systems. The data rely on CHWs visiting and being visited by members of the village in which they live. If some CHWs collect data more efficiently or effectively than others, then it will appear as if those locations have higher caseloads. If CHWs systematically exclude certain households due to geographic distance, social dynamics (e.g. caste, socioeconomic status, etc.), or interpersonal difficulties then the data may not be representative of the catchment area population. Similarly, due to significant geographic and financial barriers limiting hospital access, the cases seen at this level may be representative primarily of the nearest, most financially secure households. The community health dataset only contains data from one year of observation, which may not be long enough to validly evaluate seasonal trends compared to the hospital data. However, the modest agreement between these two sources is suggestive of CHW surveillance sensitivity to changes in disease burden in the catchment area population.

Finally, both diarrhea and ARIs are non-specific conditions that reflect diagnoses made by CHWs who are lay healthcare workers with modest training and infrequent supervision. We cannot determine from the community-level data alone the definitive diagnoses CHWs describe. Inaccurate diagnosis or coding may change the way we view the situation in unforeseen ways and may make these data inaccurate.

Despite these limitations, our study indicates that mobile phones may be a viable platform for gathering healthcare data to sustain robust community-based surveillance systems. Future studies comparing routine data collection by CHWs with independently collected village or household level rates would be valuable. It will also be valuable to more closely evaluate the differences between patients that are observed by such a mobile phone-based surveillance system. There may be differences between who is observed and who chooses to report to the CHWs based on gender, socioeconomic status, disability, or other characteristics. The role of geographic and environmental factors would also be useful to evaluate if there is any correlation between weather patterns and the trends in reported conditions, or if distance from the referral hospital plays a role in actual service utilization. Additionally, it would be valuable to follow a similar pilot design over a longer period of time to see if the CHWs significantly impact the utilization of necessary care by community members through regular patient engagement.

## Conclusions

As CHW programs expand globally, and as mobile applications for CHWs become more widespread, the volume of robust, real-time data that they collect will increase [41]. There are limited data, however, that assess the utility of such systems [42]. The findings of this study are important for similar CHW-based surveillance systems in rural resource-limited settings. Our data contribute to a proof of concept that data collected by CHWs can augment data collected at local healthcare facilities. If CHW-collected data are able to identify trends in healthcare facility data accurately and quickly, these data can be used to inform real-time decision making about resource allocation or health system preparedness for upcoming high caseloads of disease.

Despite its limitations, our analysis suggests that data collected by CHWs during their routine course of care can be used for descriptive purposes and may correlate with disease trends as seen by a corresponding district-level hospital. CHWs have the potential to bolster the regional surveillance capabilities in resource-limited settings around the world. Through providing another accurate source of data on where outbreaks of disease may be occurring, CHWs can help to inform resource allocation at local healthcare facilities. Such surveillance is critical for strengthening healthcare systems in resource limited settings around the world.
